# Surviving crack: a qualitative study of the strategies and tactics developed by Brazilian users to deal with the risks associated with the drug

**DOI:** 10.1186/1471-2458-10-671

**Published:** 2010-11-04

**Authors:** Luciana A Ribeiro, Zila M Sanchez, Solange A Nappo

**Affiliations:** 1 Universidade Federal de São Paulo/Psychobiology Department, Brazilian Center of Information of Psychotropic Drugs (CEBRID), Rua Napoleão de Barros, 925 - Vila Clementino, São Paulo, Brazil

## Abstract

**Background:**

Due to marginalization, trafficking violence, conflicts with the police and organic and social psychological problems associated with the drug, crack is one of the most devastating drugs currently in use. However, there is evidence that some users manage to stay alive and active while using crack cocaine for many years, despite the numerous adversities and risks involved with this behavior. In this context, the aim of the present study was to identify the strategies and tactics developed by crack users to deal with the risks associated with the culture of use by examining the survival strategies employed by long-term users.

**Method:**

A qualitative research method was used involving semi-structured, in-depth interviews. Twenty-eight crack users fulfilling a pre-defined enrollment criterion were interviewed. This criterion was defined as the long-term use of crack (i.e., at least four years). The sample was selected using information provided by key informants and distributed across eight different supply chains. The interviews were literally transcribed and analyzed via content analysis techniques using NVivo-8 software.

**Results:**

There was diversity in the sample with regard to economic and education levels. The average duration of crack use was 11.5 years. Respondents believed that the greatest risks of crack dependence were related to the drug's psychological effects (e.g., cravings and transient paranoid symptoms) and those arising from its illegality (e.g., clashes with the police and trafficking). Protection strategies focused on the control of the psychological effects, primarily through the consumption of alcohol and marijuana. To address the illegality of the drug, strategies were developed to deal with dealers and the police; these strategies were considered crucial for survival.

**Conclusions:**

The strategies developed by the respondents focused on trying to protect themselves. They proved generally effective, though they involved risks of triggering additional problems (e.g., other dependencies) in the long term.

## Background

Crack, the smokable form of cocaine, is a potent stimulant of the central nervous system and carries with it a high potential for user addiction. In general, crack cocaine use occurs over a prolonged period of time [1], and cessation of use results in physical, psychological or financial exhaustion [2,3].

Cocaine presents inherent risks to users, such as neurological and psychiatric impairments [4] (e.g., depression and psychosis symptoms [5,6]) and death through overdose [7].

Although it is not identified among the most heavily consumed illicit drugs from Brazilian statistical data (only 0.7% of the population has used it during their lifetime) [8], crack deserves attention due to the risks associated with the pattern of compulsive use surrounding it. Users frequently become involved in violent and illegal activities, such as theft, assault, trafficking [2,3] and risky sexual activities to obtain money or drugs [9-11], all of which cause social and public health problems.

The sharing of drug paraphernalia and sexual promiscuity lead to an increase in sexually transmitted diseases in addition to other diseases resulting from respiratory damage (e.g., tuberculosis) [12,13]. Furthermore, the lifestyle adopted by users, which is often permeated by illegal activities, makes them especially vulnerable to external causes of death [10]. In a five-year follow-up study with crack users, Ribeiro et al. showed that 18% of the sample died during the follow-up period. The authors reported that this rate is seven times greater than the general mortality rate in São Paulo during the same period and noted that 56.6% of these deaths were homicides [14].

Such facts demonstrate how destructive the culture of crack consumption can be. Due to social marginalization, violence related to trafficking, conflicts with the police and organic and social psychological problems associated with the drug, crack has become one of the most devastating drugs [2,3,10].

However, there is evidence that some users manage to stay alive and active while using crack cocaine for many years, despite the list of adversities and risks involved with this behavior. Dias et al. [15] followed crack users for 12 years and reported that deaths declined considerably during the last seven years of follow-up. They saw stabilization in deaths over time, which suggests that users learned protection strategies [15].

In a nine-year American study following crack users, Falck et al. also noted some adaptation to the crack culture [1]. They emphasized that 64% of the sample maintained unaltered use of crack for nearly a decade (i.e., use without periods of abstinence for more than six months in duration).

However, there is still a serious and important lack of understanding with regard to the practices and social dynamics related to crack consumption [16], especially among long-term users [1]. Therefore, there is a need to explore and describe in detail the strategies that seem to support this long-term crack use [2].

Trying to understand the practices and social dynamics related to crack consumption among long-term users is novel and very timely because there is a dearth of research concerning long-term users of illicit drugs, especially non-treatment populations. Such understanding may substantially subsidize the policies directed to this population.

Therefore, the objective of this study was to identify, from the perspective of the user, the risks to which users are subjected. This study then sought to explore strategies and tactics employed by users to overcome these risks and increase the chances for survival within this context.

## Methodos

A qualitative research methodology was used to describe and analyze human behavior and culture from the point-of-view of the subject investigated [17] This method permits the identification of beliefs and behavior using the subjects' own conceptual framework [18,19].

### Sample and Recruitment

One of the first stages of the study was the selection of eight key informants - people with extensive knowledge of both the topic and the study population [17]. The key informants were invited for an interview that addressed issues related to the research topic. The interviews were taped and transcribed for further analysis. The eight key informants consisted of five university professors who work in the area of abuse and drug addiction, a professional with experience in public policy and harm reduction, a current crack user and a former crack user.

The interviews provided support for the preparation of an interview guide used to collect data from the interviews with the research subjects (crack users) [19]. Due to the characteristics of the population, which usually remain hidden because of the illegality of drug use, the key informants often acted as gatekeepers, facilitating the interaction of the researchers with this population [17]. This approach enhanced the responsiveness of the population and the first contact with potential research subjects. Each of the key informants indicated potential subjects and discussed the study with them prior to introductions with the researchers. Those individuals who decided to be interviewed were told to contact the researchers.

In this way, in-depth interviews were conducted with purposeful sampling for subjects meeting the following criterion (criterion sampling [19]): subjects with experience in crack dependence defined as the use of crack for at least four years. This time was considered sufficient to cover the information-rich crack culture cases, considering the low life expectancy of users as described in first-generation studies in Brazil [3,20]. This criterion led to the final sample size of 28 interviewees, all selected in São Paulo during the years 2008-09.

The first interviewees, who were contacted by the key informants [17,21], successively recommended others, allowing for the collection of subjects via the "snowball" technique" [22]. Sampling by a snowball through a first frame (first respondent) creates a chain of respondents. The advantage of this method is that the first respondent presents people with the necessary characteristics for the study, allowing access to a hidden population that would not be accessible otherwise [22]. To cover the largest possible number of user profiles and to meet all the criteria proposed for the initial investigation, the largest possible number of different chains of users was sought. From this process, eight chains, with a maximum of five respondents in each, were identified.

The sample size (28) was sufficient to cover all topics of interest and different user profiles (gender, duration of crack use and social class). This information was detected when the interviewees eventually became redundant, as demonstrated by the lack of new information and repetition of speech, reaching a point of theoretical saturation [17,19].

### Instruments used

Semi-structured interviews were conducted in detail based on a script composed with the aid of key informants [19]. The script contained some previously standardized questions and others developed during the interview. The standardized questions were intended to facilitate the comparability of responses and reduce the interference of the interviewer. The other questions arose to clarify particular topics in each interview, allowing further development to improve understanding of the research issue [17,21].

The script addressed socio-demographic data, drug consumption history, risks arising from the use of crack and strategies developed to deal with the risks. The questions relating to the socioeconomic data were evaluated using the *Brazilian Economic Classification Criteria 2008 *scale, published by the ABEP (Brazilian Association of Research Companies-Associação Brasileira de Empresas de Pesquisa) [23]. This scale mainly considers the consumer goods possessed by the family and classifies respondents into classes A1, A2, B1, B2, C, D and E (A1 is the category with the greatest ownership, whereas E delineates a lack of ownership and includes the homeless).

Most of the data collection took place in the Department of Psychobiology of UNIFESP. An audio soundtrack of the interviews was recorded based on previously established guidelines for the qualitative technique [21] after informed consent was obtained from all participants. The interviews lasted an average of ninety minutes, and respondents were remunerated at the end of the investigation (R$ 30.00).

### Qualitative Content Analysis

Each interview was identified by an alphanumeric code comprised of the first letter of the interviewee's name, age and gender. The audio recording was transcribed verbatim and reviewed by the principal investigator. Subsequently, interviews were analyzed using the content analysis technique with Bardin's theoretical framework. The interviewees were sorted into major themes (i.e., portions in agreement with each theme) and grouped into reports [24]. At this stage, the computer software NVivo version 8 was used [25]. This program allowed greater consistency in the analysis of the qualitative data and facilitated the storage of transcribed material and the organization and codification of the interviews [19].

The themes identified were analyzed to provide meaning, taking into consideration the emic approach [24]. This step, defined as categorization, was performed by three researchers working together to add consistency and coherence to the analysis [19]. Thus, interpretations and inferences were initiated along with the offering of explanations and generation of conclusions [19].

This study was approved by the Ethics Committee of the Universidade Federal de São Paulo (n. 0552/08).

## Results

### Sample description

We interviewed 28 crack users. The sample was mainly composed of men (n = 20), with an average age of 32 years (ranging between 20 and 47). The sample's average duration of crack use was 11.5 years, with a minimum of 4 years and a maximum of 20 years. The majority of the sample was unemployed or performed some type of informal work. There was a concentration in economic class E, although the sample ranged between D and A2. Education levels ranged from incomplete elementary education to completed advanced degrees, but the sample was concentrated at the lowest education levels. The predominant civil status was separated. The sample composition can be seen in Table 1:

**Table 1:** Sample composition

*Sample description*	N
**Gender**	
Male	20
Female	8
**Age**	
20-29	10
30-39	13
40-47	5
**Duration of crack use (years)**	
4-9	7
10-15	13
16-20	8
**Work**	
Unemployed	11
Informal work	12
Working	5
Economic class*	
A or B	2
C or D	11
E	15
**Education levels**	
Incomplete elementary	9
Complete elementary	6
Incomplete high school	3
Complete high school	6
Incomplete college	3
Complete college	1
**Civil status**	
Single	8
Married	4
**Separated**	16

*This scale mainly considers the consumer goods possessed by the family and classifies respondents into classes A1, A2, B1, B2, C, D and E (A1 is the category with the greatest ownership, whereas E delineates a lack of ownership and includes the homeless).

### Risks arising from the use of crack

Crack users' perceptions of the inherent risks of drug use and consequent possibility of death due to use could be classified into three categories: risks associated with the psychological effects of the drug, overdose risk and risks due to the illegality of the drug.

#### Risks arising from the psychological effects of the drug

The risks due to the psychological effects of the drug were particularly related to cravings and transient paranoia symptoms. In these cases, the risk was not the psychological effect itself, but rather the behaviors adopted in the presence of these symptoms and their possible repercussions. As a result of a craving, the user develops a pattern of compulsive use and, therefore, begins looking for strategies, which often involve high-risk situations, to maintain drug consumption. The reported risks associated with cravings were physical injury and risky sexual behavior.

Physical injuries were a result of increased aggressiveness in the presence of a craving. Subjects reported that the intense and constant desire for crack generates a noticeable increase in aggression, promoting conflicts over drug rationing (either because one user got more than another user or because of distrust of companions). Diverse forms of aggression were demonstrated, ranging from punches, slaps and kicks to fights involving serious assaults (e.g., clubbings, beatings with a broom and stabbings). In situations of increased aggression, there was also a direct contribution of paranoid symptoms. The latter, characterized mainly by ideas and persecutory delusions, eventually culminated in fighting due to impaired judgment of reality.

*"I am a person who is somewhat distrustful of things. I use, and I think the person will do something; I transform. I become somewhat aggressive and such." *D29M

Various distortions of reality related to paranoid symptoms, such as the risk of severe physical injuries that can lead to death, were also reported. This risk is heightened when the user adopts atypical behaviors, such as attempting flight, to get rid of the hallucinations/delusions:

"I've jumped from the Minhocão [viaduct], thinking that someone was behind me. I fractured both ankles, both heels and three vertebrae of the spine. I looked like a turtle." J28M

A second set of risks also due to cravings was related to the incessant search for money to buy crack. According to reports, crack users generally become unemployed after a few months of drug use. This usually occurs because the user fails to meet schedules, loses interest in complying with rules and often appears "delusional" at work. About half of the current sample left or was fired from a regular job due to crack consumption. Given the lack of financial resources resulting from unemployment, the user commonly resorts to unlawful and risky practices (e.g., robberies, thefts and sexual activities in exchange for drugs or money) to generate income.

The modality most frequently used by women to obtain the resource was prostitution, although this was also observed in some men in the sample. In these cases, subjects noted the imminent risk of infection by an STD (sexually transmitted disease) or AIDS.

#### Overdose risk

Questions concerning organic risks were included in the questionnaire at the suggestion of the health professionals interviewed as key informants because they judged these as the most serious risks associated with crack consumption. From the perspective of those interviewed, however, this category barely existed. For example, only four respondents mentioned the possibility of drug overdose.

In fact, a minority of the sample reported that they had experienced an overdose (three interviewees). This was defined as an episode resulting in some type of hospital care (i.e., an episode that triggered effects perceived as extremely serious). Still, some of these reports of overdose could be due to the comorbid use of alcohol and crack.

"About three times (episodes of overdose), with crack and rum. I began to feel shortness of breath on the street, and my tongue began to roll, and when I got in the ambulance, I started to beat myself." E20F

About a third of the sample reported that they *had "felt sick" *or *"had almost overdosed" *on crack. The most frequent reports of *"*near *overdoses" *were *"rolling of the tongue," "obscured vision" *or alterations in heartbeat. The depth of the draw on the pipe and many days of continuous use were considered primarily responsible for these effects. An example of one such episode follows:

*"Yeah, I almost had an overdose. Once, my heart almost stopped beating. I almost died. I took "a hit*^*1"*^*; it was very strong, and a lot of smoke came out, and it was a very big stone. I got dizzy, fell to the floor, my heart accelerated and I started getting dizzy. I could not see anything; I began to tremble, to tingle everywhere." *P34M

#### Risks due to the illegality of the drug

The third category of identified risks included those associated with the illegality of the drug and, consequently, the presence of trafficking. These risks are mainly associated with violence in the *"bocada*" (locale of the drug sale) and the vicinity, caused by both drug gangs and the police. Violence in the *"bocada" *was identified as one of the main risks of crack use, and it reportedly intensifies when local trafficking laws are not enforced. The unpaid debts of crack users to dealers were reported to be the main risk factors for violent death in this context.

"And they killed my brother because he owed some crack to the dude." R25M

Stealing near the *"bocada" *and using the drug in this environment were also identified as sources of confusion with the dealer. This behavior may draw the attention of the police, which may undermine local dynamics. Thus, stealing or using crack in the buying environment also results in punishment, often in the form of physical violence.

The questions related to the police reveal a close relationship between violence and trafficking. This interaction arises due to the fear of either being approached by the police while carrying crack or being forced to reveal the locale of a drug purchase, which could culminate in the death of users due to reprisal from dealers. Episodes of violence were reported to result from conflict between users and the police, and they intensified when users tried to deny the use of crack.

### Strategies and tactics developed by users

Survival strategies and tactics used by respondents were intended to protect the users from risks or, if avoidance was impossible, at least to mitigate their consequences. Some strategies were used for a variety of risks, whereas others addressed a specific risk.

#### Use in group vs. use alone

Crack use within a group has been reported as a way to address the fears arising from auditory/visual disturbances or to obtain help with possible episodes of overdose by making available the assistance of using companions. The fear of being approached by the police and not having someone to share the problem also influences the decision to share use with others.

On the other hand, some respondents pointed out that one strategy for dealing with the possible risk of physical injury arising from discussions and fights caused by cravings and paranoia would be to use the drug alone without company. This strategy was associated with the fear of violence in a group due to increased aggressiveness. This is reflected in the following statement:

*"[I prefer to use] alone, because other people may be very crazy, at the time being with a knife in hand or something and stab me." *R25M

Subjects reported that using the drug alone would avoid incentives to steal and risks of this illegal activity. They reported that groups' presence in notorious locations for crack use indicated the presence of the drug, increasing the risk of police approaches.

#### Using the drug in protected places

Use of protected sites was described as a strategy to reduce the risk of violence and injuries. Using crack at either a subject's own home or a peer's home was the most common option. Hotels, especially in the central region of the city of São Paulo (Cracolândia, or "Crack Land"), were also common sites. Proximity to available crack, prostitution rooted in this region and "acceptance" of the practice by those involved, such as those responsible for the hotels, seem to facilitate use.

"I went into the hotel. There was already a girl with a pip.... There is one hotel dedicated to the addict, 5 Reais for half an hour. There is a hotel that is 7 Reais an hour." M34M

#### Marijuana and alcohol association

The use of other psychoactive substances as a strategy to deal with the effects of crack was clearly demonstrated by both the number of subjects reporting it and the variety of risks it was used to control. The combination of marijuana with crack use emerged as a primary strategy for the reduction of cravings, either by mixed use (cigarette of marijuana and crack) or by the use of marijuana after crack (more frequent in the sample). Mixed use arose to replace the use of pure crack. Users reported that the use of a stone in its pure form generated a compulsive pattern of consumption and a craving more intense than that generated by use of the mixed form. The following passage exemplifies this concept:

"Secure, secure (the craving). You take a stone and wrap it in the mixture you smoke three times (it takes longer to smoke). With the stone, you smoke quickly in a matter of a minute and already want another." J31M

However, it was also shown that the pattern of mixed use was sometimes very similar to that of pure crack. The use of marijuana generally continued even in the abstinence from crack and became, for some respondents, an associated dependency.

In this direction, alcohol formed the main strategy to alleviate transient paranoid symptoms, particularly the fear and aggression resulting from drug use.

"[One needs] a lot to drink to ease a little of the paranoia, which is very strong... then I thought, I already figured it all in my mind: If I took 3 rocks of crack I would have to have enough to drink for that." P34MU

The use of alcohol as a strategy to ease cravings was also reported by many respondents. They made use of this tactic to reduce the constant desire for crack use and to reduce the increased aggressiveness associated with cravings. They explained that the "calming" power of alcohol reduced their energy to search for money or drugs, thus providing possible control:

"When I saw that the drug was running out and the money was already gone, that's when I would despair. It only leaves you with one alternative: get a bottle of any liquor that you have, and drink it. At this moment, you cannot be rational; you have to take an anesthetic, something that calms you." J47M

The use of alcohol appeared in every extension of crack use in the study, and it was particularly common for the two substances to be used simultaneously; however, some statements indicated an increase in cravings due to the use of alcohol. Subjects reported that alcohol "leads to crack use" by immediately intensifying feelings of craving.

#### Where and when to buy the drug

Choosing the location and timing for buying drugs is seen by many users as a form of protection from episodes of violence involved with trafficking and the police.

The choice of purchase location seems to be influenced by whether the user is known in the *"bocada"*. Some prefer to go to known locations to avoid possible confusion with drug dealers during initial meetings, whereas others prefer to vary their purchase location to ensure anonymity. For the choice of when to buy, some respondents said that they rely on help from informants (people who know the dynamics of trafficking or dealers) to determine what time is most convenient. In this way, they aim to avoid confrontations with the police.

#### Delivery

"Delivery" is an alternative method by which users can purchase crack. This type of purchase occurs when users have enough money to pay for delivery of the drug. In this way, users protect themselves from the troubles associated with buying on the street, especially the approach of police.

"I'm simply leaving work, and I go to have a beer somewhere. I say: deliver to me in such a place. I have the convenience of having money and pay the dude to come and bring [the drug]. Why should I run the risk of being imprisoned?" H36M

#### Complying with trafficking rules

The majority of reports made it clear that trafficking rules should be rigorously followed. These rules generally regulate behavior between drug dealers and consumers. Once these rules are violated, the consequences are severe. "Do not go into debt" was the rule most often cited, followed by "do not try to negotiate with the dealer," "talk little at the time of purchase" and "never arrive changed in the *"bocada.*"

"The dude said to the dealer: after a while 'I'll bring [the money].' Then, the dude doesn't bring it, and stops buying in this place where he was owing; he starts buying in another location. Then, by chance, the dude went and said: 'Oh, the dude is spending over there.' Then the dudes went and 'waxed him' on the same day [killed him]. Debt is very dangerous... if the dude cannot afford to pay, afterwards he is hunting for [ensures] his own death." A32M

According to reports, the strategy of "don't go into debt" is extremely important for survival in the crack culture. It is known that the main risk associated with unpaid debt is the murder of the user. On this front, the information presented in the interviews was consistent about both the need to respect trafficking rules and the consequences when such rules are ignored.

#### Admitting use if approached by the police

Admitting use if approached by the police was reported as a protective measure against arrest and police violence. Hiding use or *"making fools of the police" *did not seem to be a good strategy because it created more conflict and violent repercussions. Revealing the location of the drug is seen as a good strategy to avoid the embarrassment of inspection. An example follows:

"...) If they ask, 'do you have any drugs there?' I say 'yes, I do, it's inside the packet of cigarettes that you have there, you can take out the lining that you find.' I say, 'no problem', I will not be sentenced to 15 years in prison, not even a year, and I will not even take a shot because of that or a slap in the face." J47M

After admission of use, some respondents reported that certain police would try to counsel them and encourage the cessation of use.

#### Condom use

Condom use was reported by few users as an essential strategy to deal with the risks of prostitution. The use of condoms was presented as occasional for most subjects (i.e., occurring when they had a condom or when the partner was unknown) and was also considered unnecessary by some.

"I wore a condom. Not always, but I did. It wasn't with everyone because (with) those that I already knew who they were, I didn't wear one." A21F

#### Strategies for keeping job ties

Some users choose to be self-employed, either by finding work that does not require the maintenance of fixed hours or by developing some kind of informal work (e.g., watching cars on the street). Such users choose to use crack on days that they will not work, or they choose to work on days when they have not used the day before, as in the following example:

"So I won't use during this week; I'll leave it until next week to use when I want to take time off. I had to use enough to be able to stop for a little time to work and do my things because it [the stone] does not allow you to work." J47M

## Discussion

Given the high mortality rate among the population of crack users [14,15] and, paradoxically, the long-term consumption for most users who remain alive [1], the question arises: "How are some users able to survive this culture and continue using for decades?"

This type of question had not been explored previously in literature through the vision of crack users. Despite this, some reports have shown that changes in the crack culture may have contributed, in some cases, to an increase in the life expectancy of consumers. In the early 1990s, it was estimated that a crack consumer would live for just a few years [3,26]. Today, it is common to find users with more than five years of consumption history [14]. An example of this finding is the sample in this study, which demonstrated a surprisingly high average time of crack use (11.5 years). This finding parallels a U.S. study that also showed the continuing use of crack for years and even decades [1].

The results of this study confirm the hypothesis that crack users "adapt" to the drug culture and some of the risks it generates. The latter, which are well-perceived by users, are confirmed by their detailed reports.

Apparently, some strategies and tactics are effective for maintaining and improving the dynamics of users' lives. In particular, effective strategies enable the user to recognize situations of greatest risk, generally, those arising from the illegality of the drug and its psychological effects, and to learn to deal with them. Because these strategies were born inside the crack culture itself and were developed by the consumers themselves, they seem to have been more easily absorbed by the culture. In a study conducted in Canada, Boyd et al. [27] concluded that measures of harm reduction are more effective when passed through the practices of current users and their associates. In the same way, Sherman et al. [28] showed the importance of peer diffusion of prevention information among drug injectors, facilitating the accessibility and reducing the stigma.

Death arose as the inherent risk in crack use that was most feared by users. Avoidance of this risk allowed the maintenance of addiction for many years. Most of the cited survival strategies were aimed at circumventing possible death and maintaining use in conditions that satisfied the dependence of the respondent.

Ribeiro et al. [14] presented homicide as the leading cause of death among crack users. Along this line, issues related to the illegality of the drug (e.g., trafficking and police) were emphasized in the present study. Haasen and Krausz [29] also claimed that crack-related homicides were directly related to the risks posed by the illicit drug market. Strategies described to facilitate dealer-user relations, which are underdeveloped relative to the risks to which they are intended, often seem to prevent serious injuries. Not challenging the dealer on apparently simple rules (e.g., not trying to negotiate price, paying debts, not causing problems around the *"bocada" *to arouse the attention of the police, choosing an appropriate place of purchase or soliciting the drug through a delivery service) can prevent the death of the user.

Additionally, regarding the illegality of the drug, the literature and the present data show that the treatment of this population by the police is not cordial [30]. The most useful strategy reported by users in this context was to admit use to the police if approached. This strategy led to a decrease in violence, as the police are more lenient with simple drug users than with those suspected of being dealers. This attitude is based upon a change in drug laws in the country, which decriminalized the behavior of users to dispense with the need for consumers caught with drugs to be taken to the police station (Law 11343/2006) [31]. Ensuring placement in the 'user' rather than the 'dealer' category was noted as a beneficial strategy to avoid police violence or punishment.

Due to police access in this population, intervention programs should include police officers playing an educational-preventative role. In this context, Malchy et al. [16] also suggested that policies for the "street" must be based on realistic programs for the care of this population.

In another group of reported survival strategies, the use of other psychoactive substances to reduce cravings led to a series of additional risks. The strategy of additional substance use contradicts previous reports in which users report avoiding the use of other substances [2] because, according to the subjects in those previous reports, such a strategy would be suboptimal because it would reduce or modify the positive effects of crack [2]. In the reports from this study, however, subjects reported that the use of other drugs, especially alcohol and marijuana, helped to alleviate the negative effects of crack use, primarily cravings, withdrawal symptoms and unwanted side effects.

In agreement with the present study, Magura and Rosenblum [32] observed that 60% of cocaine users frequently used alcohol to relieve discomfort related to cravings and the cessation of use. The literature suggests that this association aims to alleviate discomfort, especially during periods of abstinence [32]. In the present study, the use of alcohol was observed at various times, even simultaneously with crack, for a variety of purposes, many of which centered upon the reduction of unpleasant psychological effects.

The use of alcohol, reported as effective in minimizing the principle unpleasant psychological events (i.e., craving and paranoid symptoms), would help reduce risks related to behavioral conduct adopted in the presence of these symptoms. Thus, it is likely that this association plays a role in the survival of users while confronting these risks, but further studies are needed to examine this question in depth. This association raises concern due to its vast short- and long-term consequences, such as the development of another associated dependency. Moreover, the formation of the metabolite cocaethylene is also a concern. This product of combined ingestion, whose half-life is three times that of cocaine, apparently promotes an extension of the pleasurable effects for the user and results in significant stimulation. In addition, cocaethylene has properties that are more cardiotoxic than crack, such as increased heart rate and blood pressure, and it can increase the risk of an overdose [33-35].

The consumption of marijuana along with crack as a strategy has also been associated with various outcomes. The main effect presented here was the relief of cravings. Despite the existence of reports that marijuana induces cravings and encourages the development of compulsive mixed use, several reports have attributed success to this association. Labigalini et al. [36] reported successful experiences with crack users in which they could replace crack with marijuana over a reasonable period of time. As dependence on marijuana is much less damaging than dependence on crack, these authors suggest that this strategy be considered to reduce damage.

This association appears to be more common than originally thought. Therefore, further study with regard to both the results it can produce and its medium- and long-term disadvantages is needed.

Other strategies presented in the reports (e.g., using the drug in groups), however, were entirely harmful to the users. This strategy, which was reported by users as a way to protect against possible episodes of overdose due to the potential for immediate assistance, also potentially increases the chances of violent injury. Reports have indicated that there is considerable confusion among members using in group settings related to persecutory delusions and cravings during collective use. These events trigger physical confrontations between users. In addition, Latkin et al. suggested that the drug-use network influences behavior in relation to consumption; it increases drug use, causing greater susceptibility to overdosing and sharing of paraphernalia [37].

With regard to episodes of overdose, little research has examined the prevalence of this event for cocaine and its derivatives. Further, neither social nor contextual factors associated with overdosing have been considered [37]. Mesquita et al. showed that 20% of 396 exclusive cocaine users surveyed had suffered one or more episodes of this nature, with 50% of the sample knowing of one or more cases of death by overdose [38]. The authors believe that overdose events are under-reported, probably because users do not wish to present themselves as such [38].

In this study, a minority of the sample reported experiencing this event or witnessing or hearing news of a death due to such circumstances. A considerable portion of respondents reported having "felt sick" when smoking crack, with the reported symptoms clearly characterizing episodes of overdose. Thus, while overdose events may be underestimated, this underestimation likely occurs because such events are not identified as overdoses due to the fear of self-exposure.

Regarding HIV, this infection is more prevalent among crack users compared to the general population [9]. The statistics describing HIV as the second most prevalent cause of death among crack users [14] are explained by some factors related to the lifestyle of users. Apart from the vast literature on the risky sexual behavior adopted by crack users (particularly sexual activity to obtain money or drugs) [9,11,39], this study has shown that users consider condom use to be optional. Thus, the use of condoms is inconsistent. This strategy protects few users and does not prevent infection and consequent death due to HIV for the majority of users. Friedman et al., in a study with long-term injection drug users, described that some injectors remain uninfected by HIV and HCV [40]. They use personal strategies and tactics developed to stay safe [40]. The authors also showed that multiple intentionalities are integrated to keep the user uninfected [40].

The present study detected that inconsistent behavior among users extends to many survival strategies and the evaluation of risks. Some users exhibit a deficit in the ability to recognize or judge potential risks. This would make them unlikely to learn and adopt strategies to deal with such risks. These users may represent those most at risk for a poor prognosis (e.g., STDs or death). Therefore, it is possible to observe how prevention strategies need to be interconnected with objectives that cover different user behaviors.

This qualitative study was designed based on intentional sampling criteria; therefore, the ability to extrapolate these findings to other populations or to represent the standardized behavior of the general population of crack users remains limited.

## Conclusions

The results of the present study show that important changes in the crack culture, primarily related to the increased life expectancy of the user, are closely related to the adaptation of the user to this culture. Identifying key risks and developing empirical strategies to deal with these risks seem to be the key to user survival. Episodes resulting from the psychological effects and the illegality of the drug were the principle risks reported. Strategies that facilitate the user's understanding of issues relating to the illicit sale of drugs have a decisive role in minimizing episodes of violence and death.

It should be noted, however, that some strategies may have an important short-term role. As in the case of alcohol association, such strategies may have destructive long-term consequences (e.g., the addition of other dependencies). Thus, we suggest that future studies explore the specificities of each strategy and investigate the feasibility of extrapolating these strategies to intervention programs for the population of crack users.

## Competing interests

The authors declare that they have no competing interests.

## Authors' contributions

LAR managed the data collection, conducted preliminary data analysis and drafted the manuscript. ZMS conducted the final data analysis and revised the manuscript. SAN designed the research questions and was responsible for general coordination and revised the manuscript. All authors read and approved the final manuscript.

## Pre-publication history

The pre-publication history for this paper can be accessed here:

http://www.biomedcentral.com/1471-2458/10/671/prepub

## References

[B1] Falck RF Wang J Carlson RG Crack cocaine trajectories among users in a Midwestern American city Addiction 2007 102 1421 1431 10.1111/j.1360-0443.2007.01915.x 17645426

[B2] Oliveira LG Nappo SA Caracterização da cultura de crack na cidade de São Paulo: padrão de uso controlado Rev Saude Publica 2008 42 4 664 671 18641794 10.1590/s0034-89102008005000039

[B3] Nappo SA Galduróz JC Noto AR Crack Use in São Paulo Subst Use Misuse 1996 31 5 565 579 10.3109/10826089609045827 8777739

[B4] Oliveira LG Barroso LP Silveira CM Sanchez ZVM Carvalho Ponce J Vaz LJ Nappo SA Neuropsychological Assessment of Current and Past Crack Cocaine Users Subst Use Misuse 2009 44 1941 1957 10.3109/10826080902848897 20001290

[B5] Zule WA Morgan-Lopez AA Lam WKK Wechsberg WM Luseno WK Young SK Perceived Neighborhood Safety and Depressive Symptoms Among African American Crack Users Subst Use Misuse 2008 43 445 468 10.1080/10826080701203054 18365943

[B6] Smith MJ Thirthalli J Abdallah AB Murray RM Cottler LB Prevalence of psychotic symptoms in substance users: a comparison across substances Compr Psychiatry 2009 50 245 250 10.1016/j.comppsych.2008.07.009 19374969 PMC2743957

[B7] Silva EA Noto AR Formigoni MLOS Death by drug overdose: impact on families J Psychoactive Drugs 2007 39 3 301 306 18159785 10.1080/02791072.2007.10400618

[B8] Carlini EA Galduroz JCF Silva AAB Noto AR Fonseca AM Carlini CM Oliveira LG II Levantamento Domiciliar sobre o uso de drogas psicotrópicas no Brasil: estudo envolvendo as 108 maiores cidades do Brasil, 2005 2007 CEBRID/SENAD

[B9] Azevedo RCS Botega NJ Guimarães LAM Crack users, sexual behavior and risk of HIV infection Rev Bras Psiquiatr 2007 29 1 26 30 17435924

[B10] Duailibi LB Ribeiro M Laranjeira R Profile of cocaine and crack users in Brazil Cad Saude Publica 2008 24 Suppl 4 s545 557 18797730 10.1590/s0102-311x2008001600007

[B11] Nappo SA Sanchez ZVM Oliveira LG Crack, AIDS, and women in São Paulo, Brazil Subst Use Misuse 2010 45 1008 10.3109/10826084.2010.503480 20735215

[B12] Fischer B Rehm J Patra J Kalousek K Haydon E Tyndall M Crack across Canada: comparing crack users and crack non-users in a Canadian multi-city cohort of illicit opioid users Addiction 2006 101 1760 1770 10.1111/j.1360-0443.2006.01614.x 17156175

[B13] Story A Bothamley G Hayward A Crack Cocaine and Infectious Tuberculosis Emerg Infect Dis 2008 14 9 1466 1469 10.3201/eid1409.070654 18760022 PMC2603115

[B14] Ribeiro M Dunn J Sesso R Dias AC Laranjeira R Causes of death among crack cocaine users Rev Bras Psiquiatr 2006 28 3 196 202 10.1590/S1516-44462006000300010 17063219

[B15] Dias AC Ribeiro M Dunn J Sesso R Laranjeira R Follow-Up Study of Crack Cocaine Users: Situation of the Patients After 2, 5 and 12 Years Subst Abus 2008 29 3 71 79 10.1080/08897070802218125 19042208

[B16] Malchy L Bungay V Johnson J Documenting practices and perceptions of "safer" crack use: A Canadian pilot study International Journal of Drug Policy 2008 19 339 341 10.1016/j.drugpo.2007.06.005 18638705

[B17] World Health Organization Qualitative research for health programs 1994 Geneva: Division of Mental Health

[B18] Taylor SJ Bogdan R Introduction to Qualitative Research Methods 1998 New York: John Wiley & Sons Inc

[B19] Patton M Qualitative research and evaluation methods 2002 3 Thousand Oaks: Sage Publications

[B20] Laranjeira R Rassi R Dunn J Fernandes M Mitsuhiro S Crack cocaine - A Two-Year Follow-up of Treated Patientes J Addict Dis 2001 20 43 48 10.1300/J069v20n01_05 11286430

[B21] Creswell JW Research design: Qualitative, quantitative and mixed methods approaches 2009 3 USA: Sage Publications

[B22] Biernacki P Waldorf D Snowball Sampling: Problems and Techniques of Chain Referral Sampling Sociol Methods Res 1981 10 2 141 163

[B23] ABEP (Associação Brasileira de Empresas e Pesquisa) Critério de Classificação Econômica Brasil 2008

[B24] Bardin L Análise de Conteúdo 2004 3 Lisboa: Edições 70

[B25] Gibbs GR Qualitative Data Analysis: Explorations with NVivo 2009 New York: Open University Press

[B26] Nappo SA Galduróz JC Noto AR Uso do "crack" em São Paulo: fenômeno emergente? Rev ABP-APAL 1994 16 75 83

[B27] Boyd S Johnson JL Moffat B Opportunities to learn and barriers to change: crack cocaine use in the Downtown Eastside of Vancouver Harm Reduction Journal 2008 5 34 10.1186/1477-7517-5-34 19014696 PMC2613138

[B28] Sherman SG Gann DS Tobin KE Latkin CA Welsh C Bielenson P "The life they save may be mine": Diffusion of overdose prevention information from a city sponsored programme International Journal of Drug Policy 2009 20 137 142 10.1016/j.drugpo.2008.02.004 18502635

[B29] Haasen C Krausz M Myths versus Evidence with Respect to Cocaine and Crack: Learning from the US Experience Eur Addict Res 2001 7 159 160 10.1159/000050736 11752846

[B30] Raupp L Adorno RCF Circuitos de uso de crack na região central da cidade de São Paulo Cien Saude Colet 2010 http://www.abrasco.org.br/cienciaesaudecoletiva/artigos/artigo_int.php?id_artigo = 2668 Retrieved 26^th ^Dez 2009 from 10.1590/s1413-81232011000500031 21655735

[B31] Law n° http://www.planalto.gov.br/ccivil/_Ato2004-2006/2006/lei/L11343.htm 11343/2006 Retrieved 20^th ^Jan 2010 from

[B32] Bungay V Women's health and use of crack cocaine in context: Structural and 'everyday' violence International Journal of Drug Policy 2010 21 4 321 329 10.1016/j.drugpo.2009.12.008 20116989

[B33] Magura S Rosenblum A Modulating Effect of Alcohol use on cocaine use Addict Behav 2000 25 1 117 122 10.1016/S0306-4603(98)00128-2 10708326

[B34] Gossop M Manning V Ridge G Concurrent use of alcohol and cocaine: Difference in patterns of use and problems among users of crack cocaine and cocaine powder Alcohol Alcohol 2006 41 2 121 125 16455796 10.1093/alcalc/agh260

[B35] Gossop M Manning V Ridge G Concurrent use and order of use of cocaine and alcohol: behavioral differences between users of crack cocaine and cocaine powder Addiction 2006 101 1292 1298 10.1111/j.1360-0443.2006.01497.x 16911728

[B36] Labigalini E Rodrigues LR Silveira DX Therapeutic use of cannabis by crack addicts in Brazil J Psychoactive Drugs 1999 31 4 451 455 10681113 10.1080/02791072.1999.10471776

[B37] Latkin CA Hua W Tobin K Social network correlates of self-reported non-fatal overdose Drug Alcohol Depend 2007 73 61 67 10.1016/j.drugalcdep.2003.09.005 14687960

[B38] Mesquita F Kral A Reingold A Haddad I Sanches M Turienzo D Piconez D Araujo PJ Bueno R Overdoses among cocaine drug users in Brazil Addiction 2001 96 12 1809 1813 10.1046/j.1360-0443.2001.9612180910.x 11784473

[B39] Malta M Monteiro S Lima RMJ Bauken S Marco A Zuim GC HIV/AIDS risk among female sex workers who use crack in Southern Brazil Rev Saude Publica 2008 42 5 830 837 10.1590/S0034-89102008000500007 18833383

[B40] Friedman SR Mateu-Gelabert P Sandoval M Hagan H Des Jarlais DC Positive deviance control-case life history: a method to develop grounded hypotheses about successful long-term avoidance of infection BMC Public Health 2008 8 94 10.1186/1471-2458-8-94 18366699 PMC2329618

